# Gender Differences in Prevalence and Risk Factors for Hypertension among Adult Populations: A Cross-Sectional Study in Indonesia

**DOI:** 10.3390/ijerph18126259

**Published:** 2021-06-09

**Authors:** Selly Ruth Defianna, Ailiana Santosa, Ari Probandari, Fatwa Sari Tetra Dewi

**Affiliations:** 1 Faculty of Medicine, Public Health and Nursing, School of Public Health, Universitas Gadjah Mada, Yogyakarta 55281, Indonesia; sellyruth24@gmail.com; 2 Department of Public Health and Community Medicine, Institute of Medicine, Sahlgrenska Academy, University of Gothenburg, 41390 Gothenburg, Sweden; ailiana.santosa@gu.se; 3 Department of Public Health, Faculty of Medicine, Universitas Sebelas Maret, Surakarta 57126, Indonesia; ari.probandari@staff.uns.ac.id; 4 Disease Control Research Group, Faculty of Medicine, Universitas Sebelas Maret, Surakarta 57126, Indonesia; 5 Department of Health Behavior, Environment and Social Medicine, Faculty of Medicine, Public Health and Nursing, Universitas Gadjah Mada, Yogyakarta 55281, Indonesia; 6 Sleman Health Demographic and Surveillance System, Faculty of Medicine, Public Health and Nursing, Universitas Gadjah Mada, Yogyakarta 55281, Indonesia

**Keywords:** hypertension, gender, adult population, abdominal obesity, Indonesia

## Abstract

Although hypertension is among the main public health concerns in Indonesia, due to the scarcity of data, few studies have investigated the factors associated with hypertension in men and women. This study aimed to examine the prevalence of and factors associated with hypertension among adult men and women in Indonesia. The 2018 Survey of the Sleman Health Demographic and Surveillance System was utilized, consisting of 4328 individuals aged 18+ years. Multivariable logistic regression analysis was performed to determine the sociodemographic and health behavior factors of hypertension. Overall, the prevalence of hypertension was 40% (42% in men and 38% in women). Age, abdominal obesity and chronic non-communicable diseases were the common predictors of hypertension in men and women (*p* < 0.05). The odds ratio of hypertension among men with low education was lower than among those with high education (OR = 0.52, 95% CI: 0.29–0.94). For women, being in the poorest socioeconomic condition increased the risk of hypertension by 1.67 times compared to the richest (95% CI: 1.21–2.32). Gender differences in the prevalence of and factors associated with hypertension were observed among adult populations in Sleman District, Yogyakarta, Indonesia. Therefore, a gender-based approach in the health prevention strategy to control hypertension for men and women is needed.

## 1. Introduction

Non-communicable diseases (NCD) are the leading cause of death globally and have become one of the main health challenges around the world, especially in low- and middle-income countries (LMIC) [1]. In 2016, deaths due to NCD constituted 71% (41 million) of the total 57 million deaths globally [2]. Deaths caused by NCD in Indonesia in 2016 amounted to 1.35 million or 73% of the total 1.8 million deaths [1]. Indonesia has the second highest prevalence of hypertension in the Southeast Asia Region after Myanmar [3,4]. The Indonesian Basic Health Research (Riskesdas) showed a U-shaped trend of prevalence of hypertension among individuals aged 18 years and above, with a decreasing trend between 2007 and 2013 (from 31.7% to 25.8%), followed by an increase by 8.3% in 2018 [5]. A slight increase in self-reported hypertension was noticed, from 7.2% in 2007 to 8.8% in 2018. The proportion of hypertensive people being treated was still low (<0.5%) [5]. 

Gender disparities in NCD risk factors have been identified from previous studies in low- or middle-income and high-income countries [6,7,8,9]. Findings from an Indonesian national survey in 2015 showed that more women were diagnosed with hypertension than men (50.1% vs. 33.7%) [9]. Among those who were diagnosed, only around 11.5% (men 8.4% vs. women 14.0%) were being treated, and only 14.3% (men 12.4% vs. women 15.7%) had their blood pressure controlled to less than 140/90 mmHg. The Riskesdas report in 2013 showed that the prevalence of self-reported hypertension among adults in Yogyakarta was 25.7% [5]. Previous studies in Yogyakarta province revealed that risk factors for hypertension were obesity, intake of fruits and vegetables [10], physical activity and anxiety levels [11]. Several studies showed that factors associated with hypertension among the Indonesian adult population were age [12,13], low education, overweight or obesity and health care utilization in the last month [14]. Smoking cessation and depression were the factors most strongly associated with hypertension among men, while, among women, low socioeconomic status (SES) was the most important factor [14]. Based on our knowledge, there is a scarcity of research on gender differences in the determinants of hypertension in Indonesia. Moreover, to develop early prevention and effective blood pressure management programs in Indonesia, a gender-specific approach is needed. Using the secondary data from a surveillance site, Sleman Health Demographic and Surveillance System (Sleman HDSS), which was initiated in 2014 to represent the district population data [15], this current study aimed to examine gender differences in the prevalence of and risk factors associated with hypertension among adult populations in Sleman District, Yogyakarta, Indonesia.

## 2. Materials and Methods

### 2.1. Study Population and Study Design

This cross-sectional study was conducted with data from the 2018 Sleman HDSS—a longitudinal and community-based survey established in 2014 through a close collaboration between the Faculty of Medicine, Public Health and Nursing Universitas Gadjah Mada, and the Government of Sleman District, Yogyakarta Special Region, Indonesia [15,16]. Detailed information about the Sleman HDSS can be found elsewhere [15]. For the purpose of this study, we only included subjects who were aged ≥18 years and participated in the Sleman HDSS survey (*N* = 5640). The complete data for analysis included 4328 individuals, excluding those with missing data.

### 2.2. Dependent Variables

The focus of the study was the prevalence of hypertension. Blood pressure was measured using a Digital Automatic Blood Pressure Monitor HEM-7200 (Omron Healthcare Co., Ltd., Kyoto, Japan) on the right arm with the position aligned with the heart. The measurements were taken three times at home by trained enumerators, in a sitting position after rest for 15 min, with three-minute intervals for each measurement. Hypertension was defined if systolic blood pressure (SBP) ≥140 mmHg and/or diastolic blood pressure (DBP) ≥90 mm Hg or individuals taking blood pressure lowering medication [14].

### 2.3. Independent Variables

We included sociodemographic, SES, and health behavior risk factors as potential determinants for hypertension. Sociodemographic factors were derived from individual questionnaires consisting of questions about age group (18–39 year; 40–55 year; 56 year and above); location area (urban and rural area); educational level, which was subdivided into four categories—low education (never attended school and no formal schooling), primary school (elementary and junior high school), secondary school (senior high school), and diploma/undergraduate/university); and marital status, which was subdivided into three groups—married/reconciled, unmarried, and divorced/widowed/separated. The SES was derived from a number of questions on household assets and ownership. Principal component analysis (PCA) was used to construct the wealth index, which we later categorized by quintile (1st quintile refers to the poorest group and 5th quintile to the richest group) [17,18].

For behavioral risk factors, we included physical activity, smoking status, and waist circumference measurement. Smoking status was assessed with the question, “Did you smoke in the past 1 month? (1. yes, every day, 2. yes, sometimes, 3. no, but previously smoked every day, 4. no, but I have occasionally smoked before, and 5. never)”. Responses were grouped into never smoke (if the respondent answered “never”), current smoker (if the respondent answered “yes, every day” or “yes, sometimes”), and former smoker (if the respondent answered “no, but previously smoked every day” or “no, but I have occasionally smoked before”). 

Physical activity was assessed using a modified short form of the International Physical Activity Questionnaire (IPAQ) for the types and times of physical activities performed in all areas of life: work, home, and exercise. The total duration of activities was transformed to Metabolic Equivalent of Tasks (METs)-minutes and summed to gain an overall estimate of physical activity in a week, and the data were further classified into three categories based on the WHO recommendations (for at least 150 min of moderate-intensity physical activity or 75 min of vigorous-intensity physical activity or achieving at least 600 total MET-minutes) [19]. Abdominal obesity was defined based on the waist circumference measurement (for men, if waist circumference ≥90 cm, and for women, ≥80 cm). Chronic NCD was defined as the sum of chronic diseases such as stroke, angina, diabetes, and chronic obstructive pulmonary disease, which was later reclassified into none, one chronic condition, and two or more chronic conditions. 

### 2.4. Ethical Considerations

This study received ethical approval from the Medical and Health Research Ethics Committee (MHREC) from the Faculty of Medicine, Public Health, and Nursing at Universitas Gadjah Mada (KE/FK/0434/EC/2018) for routine data collection, and additional ethical consideration for secondary data utilization was received from the same committee (KE/FK/0116/EC/2020).

### 2.5. Statistical Analysis

STATA 15.0 (StataCorp, College Station, TX, USA) was used for the analysis and all analyses were stratified by gender, with a significance value of 0.05. Descriptive analysis of the respondents’ characteristics was presented as frequency distribution and percentage of each variable. Multivariate logistic regression models were used to examine the association of sociodemographic, health risk factors, and hypertension, adjusted for other potential covariates. Estimates of odds ratio (OR) and 95% confidence interval (CI) are presented in total and by gender.

## 3. Results

Table 1 shows the descriptive characteristics of the men and women who participated in the HDSS survey. Of the 4328 individuals, 61.5% were women. Overall, the majority were married, lived in urban areas, were non-smokers, were physically active, and reported no chronic diseases. Only 15% had a high level of education. The sociodemographic characteristics of HDSS respondents differed among men and women. Women were more likely to be divorced than men (19.4% vs. 8.9%), more likely to have no education (9.9% vs. 5.4%), and more likely to have abdominal obesity (66% vs. 27%). In addition, men were more likely to smoke currently than women (53% vs.0.5%) and were more likely to have a sedentary lifestyle/low physical activity (12.2% vs. 6.1%).

We found that individuals who were older, divorced/widowed/separated, had a low education level, had a sedentary lifestyle, had abdominal obesity, and had chronic NCD were more likely to have hypertension (Table 2).

Findings from the multivariate logistic regression models (Table 3) showed that being older, male, and divorced/widowed/separate, and having the poorest SES, abdominal obesity, and chronic NCD, were significantly associated with hypertension (*p* < 0.05). The odds of hypertension increased with increasing age, with an increase of almost three times higher odds among individuals aged 40–55 years (95% CI: 2.16–3.29) and five times higher odds for the older group (56 years and above) (95% CI = 4.11–6.53) compared to the younger age group (18–39 year). Men had 1.77 times higher odds of developing hypertension than women (95% CI: 1.40–2.23), and the poorest SES group had 1.41 times higher odds of developing hypertension (95% CI: 1.09–1.81) compared to the richest SES group. The odds increased by 1.29 times for those who were divorced/separated, 2.64 times for those with abdominal obesity, and 1.68 times for those with one chronic NCD. However, physical activity and living area were not significantly associated with hypertension (*p* > 0.05).

**Figure 1:**
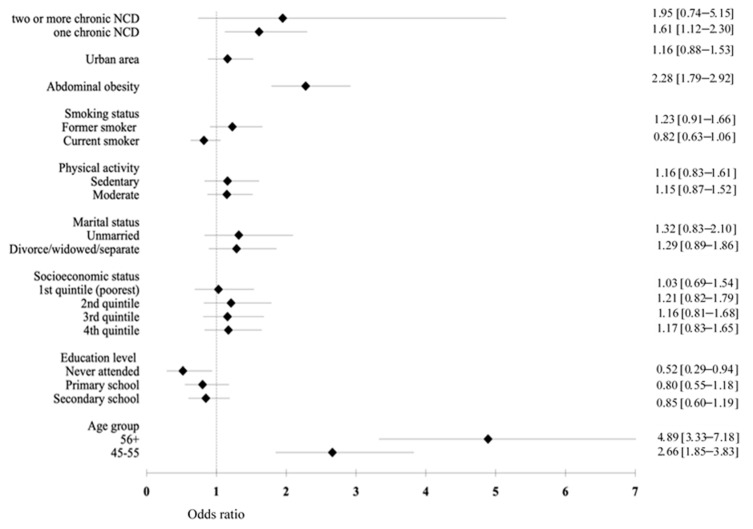
The odds of the association between sociodemographic variables, socioeconomic status, health behavior, and hypertension in men.

**Figure 2:**
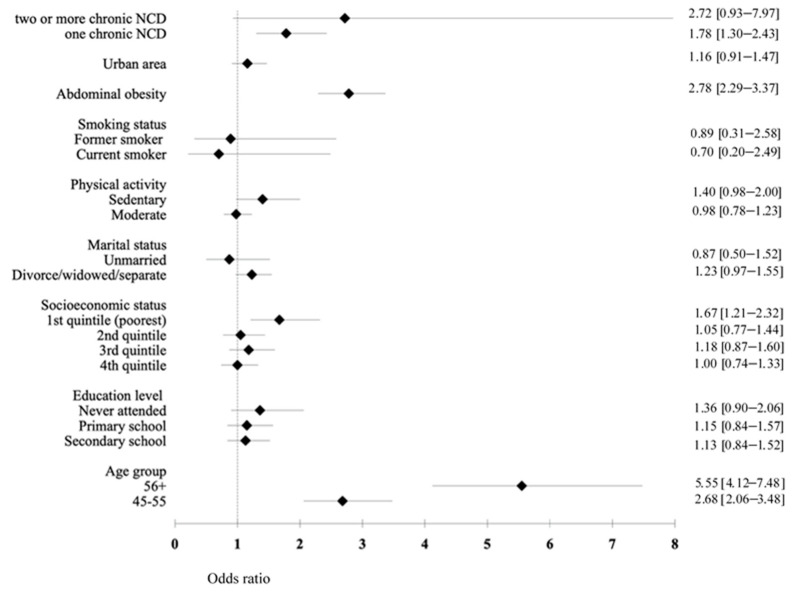
The odds of the association between sociodemographic variables, socioeconomic status, health behavior, and hypertension in women.

Findings from the multivariate regression models for men and women are displayed in Figure 1 and Figure 2. We found that the common and strong predictors of hypertension among men and women were older age, abdominal obesity, and the presence of chronic NCDs (*p* < 0.05). Men with a low education level had a lower risk for hypertension compared to the high-education groups (OR = 0.52, 95% CI: 0.29–0.94). Among women, being in the poorest SES increased the risk of hypertension by 1.67 times compared to those in the richest SES (95% CI: 1.21–2.32).

**Figure 3:**
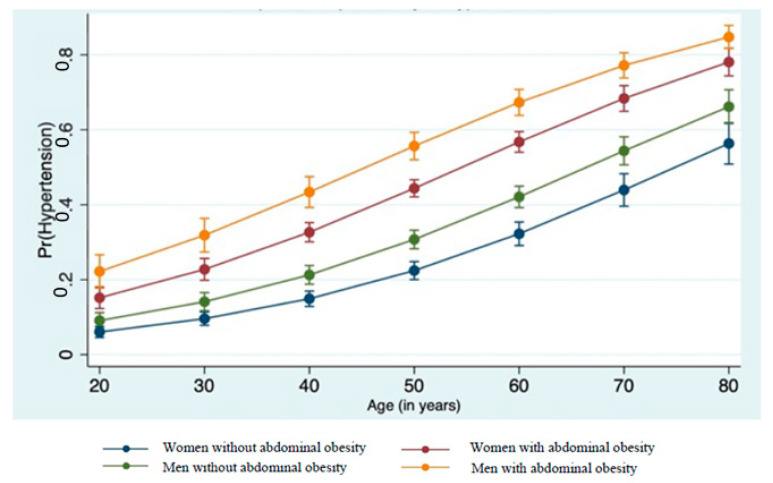
The predicted probability of hypertension, by abdominal obesity, among men and women.

We calculated the predicted probability of hypertension by abdominal obesity among men and women (Figure 3). The probability of having hypertension was predicted to increase as age increases, particularly for men with abdominal obesity.

## 4. Discussion

In the present study, we revealed gender differences in the prevalence of and factors associated with hypertension observed among adult populations in Sleman District, Yogyakarta, Indonesia. Older age, abdominal obesity, and chronic diseases were the factors most strongly associated with the prevalence of hypertension in both men and women. Socioeconomic status increased the risk of hypertension among women, while, for men, education level had the greatest impact.

### 4.1. Gender Differences and Determinants of Hypertension 

#### 4.1.1. Biomedical Factors

Abdominal central obesity is a risk factor of hypertension [19,20,21,22], which is in line with findings from the current study. All obesity measurement parameters (body mass index, central obesity, and body fat percentage) were associated with an increased likelihood of developing hypertension [23]. Abdominal obesity revealed the accumulation of abdominal fat, which implied kidney problems involving sodium reabsorption disorders and results in increasing blood pressure [20].

#### 4.1.2. Behavioral Factors

Smoking has been identified as a health risk factor for cardiovascular diseases and hypertension [24]. In this study, adult men were found to be more likely to smoke than women (52.3% vs. 0.5%, respectively). The chronic effects of habitual smoking still have not been elucidated, as the observed findings in this study showed that current smoker status was a protective risk factor of hypertension in men, which is similar to other studies [25]. A plausible explanation is that the instrument used to classify the smoking status (we only used a single question on the respondents’ smoking habit within the last month) may have led to a biased evaluation of the cumulative smoking effect on health [26]. Only measuring current smoking status may not yet show the effect of smoking on hypertension [27]. In Indonesia, having a disease is a predictor of quitting smoking, explaining why former smoker status was associated with hypertension in this study [28]. Therefore, further research is needed to investigate lifetime smoking behavior and the development of hypertension and other health outcomes.

#### 4.1.3. Sociodemographic Factors

Sociodemographic factors such as age and SES are well-known risk factors related to hypertension. A study in Indonesia showed that the risk of hypertension increases with age [13], which is in line with the findings from the current study. Additionally, men are at higher risk of hypertension, as shown in this study, which parallels similar findings from Korea [7], the US, and China [8]. 

Low SES coupled with low education levels is also associated with a high prevalence of hypertension [29]. However, in this study, we found that low education level was a significant protective factor for the development of hypertension among men, but not for women. This might be due to the interaction between those subjects with low education and the type of occupation. In our study, men with a low education level were those who were labor workers, a role that carries a high demand for physical activity. Unlike men, low SES status was a significant risk factor for hypertension among women. In fact, poor women were those who had a more sedentary lifestyle, which was observed in this study and another recent study [30]. The research location was in Yogyakarta, an area with a strong patriarchal culture, which typically assigns domestic roles to women. Economic hardship and being divorced or widowed forces poor women to play not only domestic roles but also to become the family breadwinner [31]. While the type of occupation is most likely labor workers with low physical activity, these poor women have no time left for physical activity. 

Marital status is also known to play an important role in hypertension [32]. This study found that divorced women were more likely to develop hypertension than married women, similar to studies in Ghana and Iran. In Indonesia’s context, women’s economic dependency on men drives women into poorer economic conditions after becoming divorced. This condition is aggravated by decreasing social support, worsening diet, stress, and depression because of the divorce process [32,33,34]. A national study in Indonesia found that divorced women had a negative stigma and were at higher risk of becoming trapped in poverty [35]. Many divorced women do not have legal and economic certainty, while they are still obligated to meet the economic needs of their family [32,33,34,36]. We propose that social and cultural factors may influence the prevalence of hypertension in the Indonesian context, particularly in Yogyakarta. A similar phenomenon also exists in other low- or middle-income countries [32,33,34]. Therefore, future research on cultural identification and social support is needed to gain a better understanding of these factors for more effective health promotion, prevention, and intervention programs.

### 4.2. Public Health Implications 

The gender-based approach has been considered an effective strategy for preventing and controlling NCDs [37], in line with the findings of our study. However, a systematic review found that only a few community movements applied a gender-specific approach to NCD prevention and control [38]. Hence, the problems of implementing a gender-based approach in national and local contexts should be identified and properly addressed. Continuous surveillance of gender differences in hypertension risk factors should be highlighted, simultaneously with gender-based health education. Health education is one of the best interventions for reducing tobacco use, unhealthy diets, and physical inactivity [1]. Therefore, health education among the community should be redesigned to avoid one-size-fits-all content, since the risk factors are different for men and women, as evidenced by our study findings. Nevertheless, multisectoral collaboration in policy implementation is needed to address gender differences in NCD risk factors and the importance of poverty reduction. Globally, many countries have implemented less than half of the recommended global NCD prevention and control policies [39]. In Indonesia, the “Community Movement for Health” (Germas) was implemented. However, multisectoral collaboration and support from all sectors are still formidable challenges, due to the differences in settings and the diverse priorities at the national and regional levels [40].

### 4.3. Strengths and Limitations

The strengths of the current study include utilizing HDSS data from an ongoing longitudinal survey that can provide a sampling frame for population-based studies with a high participation rate (95%). HDSS data cover individual and household information on sociodemographic variables, SES, behavioral risk factors, and chronic conditions that provide data complementary to the existing demographic and health information system at the local and national levels. Findings from this study strengthen the scientific evidence on the existing gender differences in risk factors for hypertension among adult populations in Yogyakarta, Indonesia. Some limitations of the study should be noted. The HDSS data used in the current research are from a cross-sectional study, which does not allow direct conclusions on causality to be made. Therefore, future cohort studies are needed to understand the impact of health behavior changes over time on the development of hypertension. Apart from anthropometric and blood pressure measurements, all other information provided in this study was self-reported, which could present some bias. In addition, we observed that more women participated in this study, reflecting how they might exhibit different health behaviors to men. Thus, the estimates might be over-reported or under-reported. However, in this study, we analyzed the data separately for men and women. We are also aware that some health behavior risk factors, such as stress and the consumption levels of salt, alcohol, fatty foods, and fried foods, were not considered in this study but are factors that are closely related to the outcome. Therefore, future studies exploring more health behaviors related to hypertension, as well as social factors, are needed. 

## 5. Conclusions

The prevalence of hypertension among the adult population in Sleman District, Yogyakarta, Indonesia is high, with men having a higher prevalence than women. The most common predictors of hypertension were found to be older age, abdominal obesity, and chronic diseases. Gender differences exist in the prevalence of hypertension in Indonesia, where current hypertension guidelines and management rarely focus on the gender differences. Therefore, a new hypertension control policy should adopt a gender-based approach to health education and surveillance, with strengthened input from a more proactive multisectoral collaboration.

## Figures and Tables

**Table 1:** Characteristics of HDSS respondents in 2018, in total and by gender.

Characteristics	Total. *N* (%)	Men. *N* (%)	Women *N* (%)	*p*-Value
Age group				
18–39 years	867 (20)	282 (16.9)	585 (22)	0.000
40–55 years	1881 (43.5)	696 (41.8)	1185 (44.5)	
≥56 years	1580 (36.5)	689 (41.3)	891 (33.5)	
Marital status				
Married	3469 (80.2)	1395 (83.7)	2074 (77.9)	0.000
Divorce	663 (15.3)	148 (8.9)	515 (19.4)	
Unmarried	196 (4.5)	124 (7.4)	72 (2.7)	
Education level				
University/diploma	642 (14.8)	252 (15.1)	390 (14.7)	0.000
Secondary school	1618 (37.4)	669 (40.1)	949 (35.7)	
Primary school	1715 (39.6)	656 (39.4)	1059 (39.8)	
Never attended school	353 (8.2)	90 (5.4)	263 (9.9)	
Socioeconomic status				
Q5 (richest group)	778 (18)	310 (18.6)	468 (17.6)	0.918
Q4	911 (21.1)	351 (21.1)	560 (21)	
Q3	915 (21.1)	354 (21.2)	561 (21.1)	
Q2	856 (19.8)	324 (19.4)	532 (20)	
Q1 (poorest group)	868 (20.1)	328 (19.7)	540 (20.3)	
Physical activity				
High	3197 (73.9)	1175 (70.5)	2022 (76)	0.000
Moderate	765 (17.7)	289 (17.3)	476 (17.9)	
Low	366 (8.5)	203 (12.2)	163 (6.1)	
Smoking status				
Never smoke	3036 (70.2)	403 (24.2)	2633 (99)	0.000
Current smoker	888 (20.5)	876 (52.6)	12 (0.5)	
Former smoker	404 (9.3)	388 (23.3)	16 (0.6)	
Abdominal Obesity	2197 (50.8)	446 (26.8)	1751 (65.8)	0.000
Urban area	3611 (83.4)	1370 (82.2)	2241 (84.2)	0.080
Chronic NCD				
None	3923 (90.6)	1489 (89.3)	2434 (91.5)	0.038
One chronic NCD	366 (8.5)	158 (9.5)	208 (7.8)	
Two or more chronic NCDs	39 (0.9)	20 (1.2)	19 (0.7)	
Hypertension	1705 (39.4)	692 (41.5)	1013 (38.1)	0.024

**Table 2:** Characteristics of HDSS respondents in 2018, by hypertension status.

Characteristics	Without Hypertension (*N* = 2623)	With Hypertension (*N* = 1705)	*p*-Value
Age group			
18–39 years	722 (27.5)	145 (8.5)	0.000
40–55 years	1192 (45.4)	689 (40.4)	
≥56 years	709 (27)	871 (51.1)	
Marital status			
Married	2177 (83)	1292 (75.8)	0.000
Divorced	310 (11.8)	353 (20.7)	
Unmarried	136 (5.2)	60 (3.5)	
Education level			
University	403 (15.4)	239 (14)	0.000
Secondary	1061 (40.5)	557 (32.7)	
Primary	985 (37.6)	730 (42.8)	
Never attended school	174 (6.6)	179 (10.5)	
Socioeconomic status			
Q5 (richest group)	473 (18)	305 (17.9)	0.011
Q4	566 (21.6)	345 (20.2)	
Q3	557 (21.2)	358 (21)	
Q2	544 (20.7)	312 (18.3)	
Q1 (poorest group)	483 (18.4)	385 (22.6)	
Physical activity			
High	1987 (75.8)	1210 (71)	0.000
Moderate	450 (17.2)	315 (18.5)	
Low	186 (7.1)	180 (10.6)	
Smoking status			
Never smoke	1851 (70.6)	1185 (69.5)	0.000
Current smoker	575 (21.9)	313 (18.4)	
Former smoker	197 (7.5)	207 (12.1)	
Abdominal Obesity	1151 (43.9)	1046 (61.4)	0.000
Urban area	2171 (82.8)	1440 (84.5)	0.144
Chronic NCD			
None	2454 (93.6)	1469 (86.2)	0.000
One chronic NCD	157 (6)	209 (12.3)	
Two or more chronic NCDs	12 (0.5)	27 (1.6)	

**Table 3:** The odds of the association between sociodemographic variables, SES, health behavior, and hypertension (in total).

Variables	Adjusted OR (95% CI)
Men	1.77 (1.40–2.23) ***
Age group	
40–55 years	2.67 (2.16–3.29) ***
≥56 years	5.18 (4.11–6.53) ***
Education level	
Secondary school	1.01 (0.81–1.25)
Primary school	0.99 (0.78–1.26)
Never attended school	1.06 (0.76–1.47)
Socioeconomic status	
4th quintile (richest)	1.07 (0.86–1.34)
3rd quintile	1.19 (0.95–1.50)
2nd quintile	1.12 (0.88–1.43)
1st quintile (poorest)	1.41 (1.09–1.81) **
Marital status	
Divorced/widowed/separated	1.29 (1.06–1.56) **
Unmarried	1.16 (0.82–1.64)
Physical activity	
Moderate	1.06 (0.89–1.26)
Low	1.23 (0.97–1.56)
Smoking status	
Current smoker	0.76 (0.59–0.98) *
Former smoker	1.15 (0.87–1.54)
Abdominal obesity	2.64 (2.27–3.06) ***
Urban area	1.15 (0.96–1.38)
Presence of chronic NCD	
One chronic NCD	1.68 (1.33–2.12) ***
Two or more chronic NCDs	2.31 (1.13–4.71) *

Note: Reference group were women, aged 18–39 years, university-educated, fell into the 5th quintile (richest), were married, had high physical activity, never smoked, did not have abdominal obesity, and lived in rural areas. * for *p*-value < 0.001; ** for *p*-value < 0.01, and *** for *p*-value < 0.05.

## Data Availability

Restrictions apply to the availability of these data. Data were obtained from Sleman HDSS and are available from the authors with the permission of Sleman HDSS.
